# Difference-based clustering of short time-course microarray data with replicates

**DOI:** 10.1186/1471-2105-8-253

**Published:** 2007-07-14

**Authors:** Jihoon Kim, Ju Han Kim

**Affiliations:** 1 Seoul National University Biomedical Informatics (SNUBI), Seoul National University College of Medicine, Seoul 110-799, Korea; 2 Department of Statistics, University of Wisconsin-Madison, Medical Science Center, 1300 University Ave., Madison, WI 53706, USA

## Abstract

**Background:**

There are some limitations associated with conventional clustering methods for short time-course gene expression data. The current algorithms require prior domain knowledge and do not incorporate information from replicates. Moreover, the results are not always easy to interpret biologically.

**Results:**

We propose a novel algorithm for identifying a subset of genes sharing a significant temporal expression pattern when replicates are used. Our algorithm requires no prior knowledge, instead relying on an observed statistic which is based on the first and second order differences between adjacent time-points. Here, a pattern is predefined as the sequence of symbols indicating direction and the rate of change between time-points, and each gene is assigned to a cluster whose members share a similar pattern. We evaluated the performance of our algorithm to those of K-means, Self-Organizing Map and the Short Time-series Expression Miner methods.

**Conclusions:**

Assessments using simulated and real data show that our method outperformed aforementioned algorithms. Our approach is an appropriate solution for clustering short time-course microarray data with replicates.

## Background

Time is an important factor in developmental biology, especially in dynamic genetics. For example, when a number of genes are differentially expressed under two or more conditions, it is often of great interest to know which changes are causal and which are not. When different conditions are represented by different time-points, it helps us to understand not only how a gene gets turned on or off, but also which gene-gene relationships are based on the lags in the changes. Novel genes have been identified by monitoring the transcription profiles during development [1] or by looking at the differential responses of genes under different conditions [2,3].

Conventional time-series methods are not well suited to the analysis of microarray data. Since the number of observed time-points in a microarray is usually very small, common methods such as auto-regression (AR), moving-average (MA) or Fourier analysis modeling may not be applicable. Furthermore, these classic autocorrelation approaches generate bias when applied to short time-course data [4]. In addition, observed time-points are sometimes distributed unevenly and the length of inter-time-points increases exponentially due to biological phenomena and resource limitation. For example, some commonly used time-points are 0, 4, 12, 24, and 48 hours. With conventional approaches, it is not clear how to calculate the magnitude of the slope between adjacent time-points or how to determine the window size for smoothing, when needed. In order to address these problems, clustering analysis has been widely used. Clustering algorithms, which explore the problem space whose size is the Stirling's number ∑k=1nS(n,k)
 MathType@MTEF@5@5@+=feaafiart1ev1aaatCvAUfKttLearuWrP9MDH5MBPbIqV92AaeXatLxBI9gBaebbnrfifHhDYfgasaacH8akY=wiFfYdH8Gipec8Eeeu0xXdbba9frFj0=OqFfea0dXdd9vqai=hGuQ8kuc9pgc9s8qqaq=dirpe0xb9q8qiLsFr0=vr0=vr0dc8meaabaqaciaacaGaaeqabaqabeGadaaakeaadaaeWaqaaiabdofatjabcIcaOiabd6gaUjabcYcaSiabdUgaRjabcMcaPaWcbaGaem4AaSMaeyypa0JaeGymaedabaGaemOBa4ganiabggHiLdaaaa@39ED@ where

S(n,k)=1k!∑i=0k(−1)k(ki)(k−i)nand n is the number of genes
 MathType@MTEF@5@5@+=feaafiart1ev1aaatCvAUfKttLearuWrP9MDH5MBPbIqV92AaeXatLxBI9gBaebbnrfifHhDYfgasaacH8akY=wiFfYdH8Gipec8Eeeu0xXdbba9frFj0=OqFfea0dXdd9vqai=hGuQ8kuc9pgc9s8qqaq=dirpe0xb9q8qiLsFr0=vr0=vr0dc8meaabaqaciaacaGaaeqabaqabeGadaaakeaacqWGtbWucqGGOaakcqWGUbGBcqGGSaalcqWGRbWAcqGGPaqkcqGH9aqpdaWcaaqaaiabigdaXaqaaiabdUgaRjabcgcaHaaadaaeWaqaamaabmaabaGaeyOeI0IaeGymaedacaGLOaGaayzkaaWaaWbaaSqabeaacqWGRbWAaaaabaGaemyAaKMaeyypa0JaeGimaadabaGaem4AaSganiabggHiLdGcdaqadaqaauaabeqaceaaaeaacqWGRbWAaeaacqWGPbqAaaaacaGLOaGaayzkaaWaaeWaaeaacqWGRbWAcqGHsislcqWGPbqAaiaawIcacaGLPaaadaahaaWcbeqaaiabd6gaUbaakiabbggaHjabb6gaUjabbsgaKjabbccaGiabd6gaUjabbccaGiabbMgaPjabbohaZjabbccaGiabbsha0jabbIgaOjabbwgaLjabbccaGiabb6gaUjabbwha1jabb2gaTjabbkgaIjabbwgaLjabbkhaYjabbccaGiabb+gaVjabbAgaMjabbccaGiabbEgaNjabbwgaLjabb6gaUjabbwgaLjabbohaZbaa@70BF@

in order to group similar objects together, can identify potentially meaningful relationships between objects and often their results can be visualized [5,6]. Phang *et al*. [7] devised a non-parametric clustering algorithm using only the direction of change from one time-point to the next in order to group genes in a time-course study. Ji *et al*. [8] proposed a model-based clustering method based on a hidden Markov model (HMM). These models assume that each gene expression profile has been generated by a Markov chain with a certain probability. The original dataset of *N *time-points is standardized and then transformed into a three-digit-sequence (0 = no change, 1 = up, 2 = down) with the aid of a tolerance factor. Luan *et al*. [9] used cubic splines in building a mixed-effects model, where observed time-points are treated as samples taken from underlying smooth processes. Ramoni *et al*. [10] adopted a Bayesian method for model-based clustering of gene expression dynamics. The method represents gene-expression dynamics as autoregressive equations and uses an agglomerative procedure to search for the most probable set of clusters given the available data. Wu *et al*. [11] considered a time-course gene expression dataset as a set of time series, generated by a number of stochastic processes. Each stochastic process defines a cluster and is described by an autoregressive model. A relocation-iteration algorithm is proposed to identify the model parameters and each gene is assigned to an appropriate cluster based on posterior probabilities. Ernst *et al*. [12] assigned genes probabilistically to preselected sub-patterns which were generated independent of the data in a short time-course experiment.

As this wealth of approaches shows, a lot of effort has been put into developing clustering algorithms for gene clustering; however, they have some limitations. Geneticists still need a more intuitive and statistically sound methodology. To address this issue, we propose a difference-based clustering algorithm (DIB-C) for a short time-course gene expression data. DIB-C discretizes a gene into a symbolic pattern of the first- and second-order differences representing direction and rate of change, respectively. Replicate and temporal order information from the input data are used in defining the clusters. DIB-C outputs a cross-sectional view of cluster hierarchies with varied cutoffs, shown in a 2-dimensional map for biological interpretation. The clustering procedure used by DIB-C is detailed in the Methods section.

**Figure 1:**
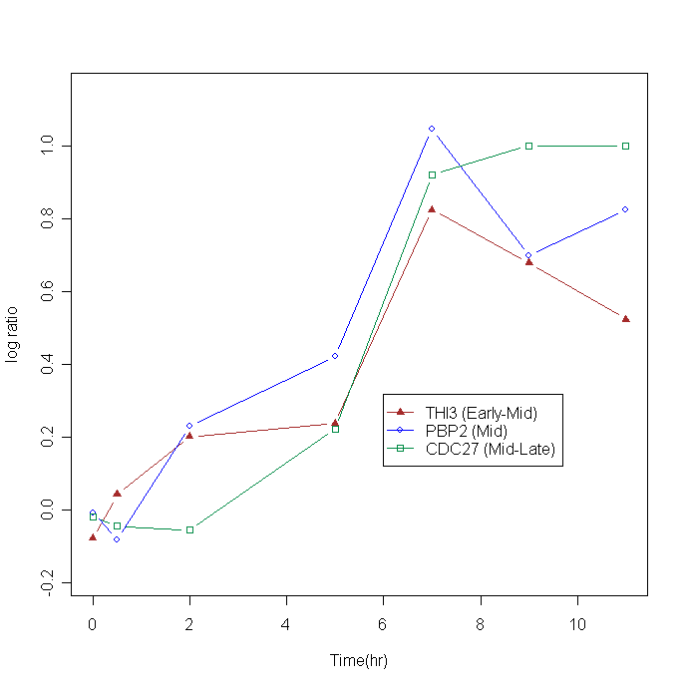
**An example of falsely clustered genes**. An example of falsely clustered genes using a conventional clustering method is shown. Three genes have similar profiles (i.e., correlation coefficients above 0.9) but the rates of change differ. The raw data were downloaded from [30].

We now examine the limitations of standard clustering algorithms and explain how we addressed each of them in developing our algorithm. First, misleading or uninterpretable clusters can occur when one only considers the similarity of expression profiles, thereby disregarding discretization information. An example from real data [1] is shown in Figure 1. The three yeast genes in Figure 1 are well studied; we know that each gene plays a different role in yeast sporulation which is characterized by sequential transcription of sets of genes-'early', 'early-mid', 'middle', 'mid-late' and 'late'. Every gene necessary for sporulation has been found to play a role in one of these five sets confirmed through genetic screens of visual assays. THI3 is known to have a specific temporal pattern in 'early-mid', PBP2 in 'mid' and CDC27 in 'mid-late' [1]. Thus, all three genes have different profiles and roles. But these three genes would have put into the same cluster if a conventional clustering method was blindly performed considering profile similarity only.

Second, the rate of change is ignored when delineating underlying patterns in traditional clustering algorithms. For example, CDC27 in Figure 1 increases from 2 hr through 11 hr, but the rate of change decreases over time. This type of saturation is often observed in biological phenomena; examples include mRNA accumulation, developmental acceleration, or gradual changes in the drug-response rate. Although some discretization-based methods which use the direction of change have been presented [7,8,13], none of these deal with differences in rates of change. We used the second-order difference – the difference between the first-order statistics – in DIB-C in order to incorporate rate of change information into the clustering procedure.

Third, replicates are not fully utilized in the existing algorithms. Kerr *et al*. [14] pointed out that replication in microarray experiments is a fundamental principle of good experimental design because it increases the precision of estimated quantities and provides information about the uncertainty of estimates. However, replicates were infrequently used in microarray experiments due to their high cost. But now, more replicates are being (and will be) used in order to achieve enough statistical power thanks to the dropping costs of microarrays with the advance of the microarray technology as in many other electronic products. Even though appropriate methods are needed to analyze this emerging type of data, most of the current methods simply compute the average over replicates, disregarding variability. In contrast, DIB-C makes use of moderated t-statistics [15], which consider an empirical Bayes variance estimate computed using the array replicates.

Fourth, conventional clustering techniques such as hierarchical clustering [16], tend to ignore temporal information by treating time-course data as an unordered collection of events under different conditions [17]. However, time-course experiments have a fixed order of conditions, *i.e*., the columns are not interchangeable [6]. The problem becomes more complicated in the presence of replicates, as any two members within-group are interchangeable unlike between-groups. DIB-C incorporates this order-restriction in the algorithm.

Fifth, template-based methods require prior knowledge to choose representative genes. In the yeast example, each gene was assigned to the nearest pre-chosen representative gene based on previous studies. Peddada *et al*. [18] also predefine a set of potential candidate profiles then assign each gene using the order-restricted inference method. However, these approaches are applicable only when there is enough information which is rare in practice.

Finally, visualization of clustering results is not always informative. *K*-means (KM) merely enumerates the list of genes, where each number signifies which cluster a gene belongs to. However, these are simply distinguishing symbols, adjacent numbers do not imply that two clusters are biologically related. Self-Organizing Map (SOM) does a little better, as it displays clustering results in a 2-dimensional grid.

## Results

We evaluated the performance of our algorithm DIB-C in comparison to *K*-means, SOM and Short Time-series Expression Miner (STEM) methods using both simulated and real data [12,19,20]. The simulation data had 19 clusters with 10 members each, at four time-points. There are eight replicates at each time point (See the Methods section for details). Real data on pancreas gene expression in mice [21] was obtained from Computational Biology and Informatics Laboratory, University of Pennsylvania [22]. We extracted 2,179 gene expression measures from a unique set of probes and used six time-points with four or six replicates at each time point. The preprocessing methods used on the pancreas data are detailed in the Methods section.

### Simulated data

For the simulated data, true clustering membership was used as knowledge external to the gene expression data. Also, the agreement between the true and the resulting cluster memberships was measured. The Adjusted Rand Index (ARI), an updated form of the Rand Index, is the number of agreements divided by the number of total objects [23] defined as:

∑i,j(nij2)−[∑i(ni.2)(n.j2)]/(n2)12[∑i(ni.2)+∑j(n.j2)]−[∑i(ni.2)∑j(n.j2)]/(n2)
 MathType@MTEF@5@5@+=feaafiart1ev1aaatCvAUfKttLearuWrP9MDH5MBPbIqV92AaeXatLxBI9gBaebbnrfifHhDYfgasaacH8akY=wiFfYdH8Gipec8Eeeu0xXdbba9frFj0=OqFfea0dXdd9vqai=hGuQ8kuc9pgc9s8qqaq=dirpe0xb9q8qiLsFr0=vr0=vr0dc8meaabaqaciaacaGaaeqabaqabeGadaaakeaadaWcaaqaamaaqababaWaaeWaaeaafaqabeGabaaabaGaemOBa42aaSbaaSqaaiabdMgaPjabdQgaQbqabaaakeaacqaIYaGmaaaacaGLOaGaayzkaaGaeyOeI0YaaSGbaeaadaWadaqaamaaqababaWaaeWaaeaafaqabeGabaaabaGaemOBa42aaSbaaSqaaiabdMgaPjabc6caUaqabaaakeaacqaIYaGmaaaacaGLOaGaayzkaaWaaeWaaeaafaqabeGabaaabaGaemOBa42aaSbaaSqaaiabc6caUiabdQgaQbqabaaakeaacqaIYaGmaaaacaGLOaGaayzkaaaaleaacqWGPbqAaeqaniabggHiLdaakiaawUfacaGLDbaaaeaadaqadaqaauaabeqaceaaaeaacqWGUbGBaeaacqaIYaGmaaaacaGLOaGaayzkaaaaaaWcbaGaemyAaKMaeiilaWIaemOAaOgabeqdcqGHris5aaGcbaWaaSGbaeaadaWcaaqaaiabigdaXaqaaiabikdaYaaadaWadaqaamaaqababaWaaeWaaeaafaqabeGabaaabaGaemOBa42aaSbaaSqaaiabdMgaPjabc6caUaqabaaakeaacqaIYaGmaaaacaGLOaGaayzkaaGaey4kaSYaaabeaeaadaqadaqaauaabeqaceaaaeaacqWGUbGBdaWgaaWcbaGaeiOla4IaemOAaOgabeaaaOqaaiabikdaYaaaaiaawIcacaGLPaaaaSqaaiabdQgaQbqab0GaeyyeIuoaaSqaaiabdMgaPbqab0GaeyyeIuoaaOGaay5waiaaw2faaiabgkHiTmaadmaabaWaaabeaeaadaqadaqaauaabeqaceaaaeaacqWGUbGBdaWgaaWcbaGaemyAaKMaeiOla4cabeaaaOqaaiabikdaYaaaaiaawIcacaGLPaaaaSqaaiabdMgaPbqab0GaeyyeIuoakmaaqababaWaaeWaaeaafaqabeGabaaabaGaemOBa42aaSbaaSqaaiabc6caUiabdQgaQbqabaaakeaacqaIYaGmaaaacaGLOaGaayzkaaaaleaacqWGQbGAaeqaniabggHiLdaakiaawUfacaGLDbaaaeaadaqadaqaauaabeqaceaaaeaacqWGUbGBaeaacqaIYaGmaaaacaGLOaGaayzkaaaaaaaaaaa@825A@

where *i *and *j *index the clusters and classes, respectively. Higher ARI values indicate more accurate clusters. The ARI is a more sensitive, generalized version of the original Rand Index and is used as our measure of comparison.

**Figure 2:**
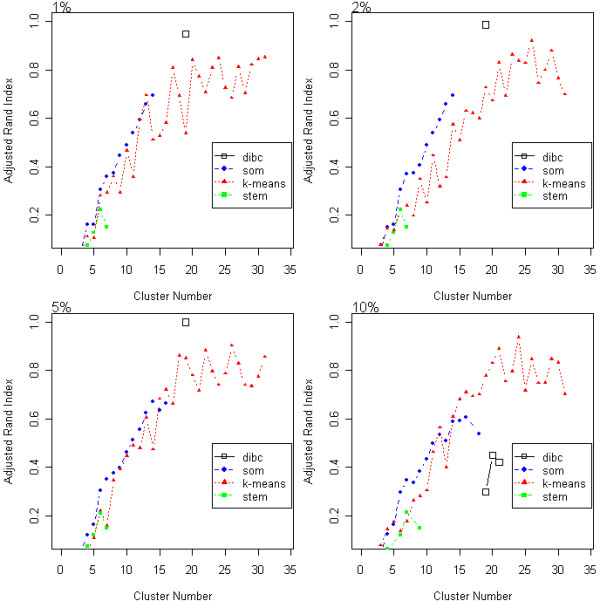
**ARI of the simulated data**. The adjusted rand index (ARI) of the simulated data is plotted according to cluster number. Higher ARI, values indicate more accurate clustering results. Three algorithms were compared under four different noise (1, 2, 5, and 10%.)

With ARI measure, DIB-C showed better accuracy across the cluster numbers than did the other three methods. Under lower noise simulations of 1, 2, and 5%, the maximum ARI values were obtained by DIB-C at the true number of clusters (19), indicating that DIB-C has the highest accuracy of the three methods (Figure 2). Under high noise (10%), *K*-means achieved the maximal ARI at 24 clusters, which was not the true cluster number. However, it is notable that DIB-C peaks at the actual cluster number. DIB-C outputs only in the neighborhood of the true cluster number unlike the other three methods because our algorithm refuses to separate insignificant changes.

As a data-driven evaluation measure without any external knowledge, the average proportion of the first eigenvalue (APF) was used to delineate the best overall clustering results. APF is the normalized proportion of eigenvalues for each cluster defined by:

ψ¯=1L∑l=1Lψlwhere,ψl=γl1∑i=1pλliL=the number of clustersP=the number of time-pointsλli=the itheigenvalue in lthcluster
 MathType@MTEF@5@5@+=feaafiart1ev1aaatCvAUfKttLearuWrP9MDH5MBPbIqV92AaeXatLxBI9gBaebbnrfifHhDYfgasaacH8akY=wiFfYdH8Gipec8Eeeu0xXdbba9frFj0=OqFfea0dXdd9vqai=hGuQ8kuc9pgc9s8qqaq=dirpe0xb9q8qiLsFr0=vr0=vr0dc8meaabaqaciaacaGaaeqabaqabeGadaaakeaafaqaaeGbcaaaaeaaiiGacuWFipqEgaqeaiabg2da9maalaaabaGaeGymaedabaGaemitaWeaamaaqadabaGae8hYdK3aaSbaaSqaaiabdYgaSbqabaaabaGaemiBaWMaeyypa0JaeGymaedabaGaemitaWeaniabggHiLdaakeaaaeaacqqG3bWDcqqGObaAcqqGLbqzcqqGYbGCcqqGLbqzcqGGSaalaeaaaeaaaeaacqWFipqEdaWgaaWcbaGaemiBaWgabeaakiabg2da9maalaaabaGae83SdC2aaSbaaSqaaiabdYgaSjabigdaXaqabaaakeaadaaeWaqaaiab=T7aSnaaBaaaleaacqWGSbaBcqWGPbqAaeqaaaqaaiabdMgaPjabg2da9iabigdaXaqaaiabdchaWbqdcqGHris5aaaaaOqaaaqaaiabbYeamjabg2da9iabbsha0jabbIgaOjabbwgaLjabbccaGiabb6gaUjabbwha1jabb2gaTjabbkgaIjabbwgaLjabbkhaYjabbccaGiabb+gaVjabbAgaMjabbccaGiabbogaJjabbYgaSjabbwha1jabbohaZjabbsha0jabbwgaLjabbkhaYjabbohaZbqaaaqaaiabbcfaqjabg2da9iabbsha0jabbIgaOjabbwgaLjabbccaGiabb6gaUjabbwha1jabb2gaTjabbkgaIjabbwgaLjabbkhaYjabbccaGiabb+gaVjabbAgaMjabbccaGiabbsha0jabbMgaPjabb2gaTjabbwgaLjabb2caTiabbchaWjabb+gaVjabbMgaPjabb6gaUjabbsha0jabbohaZbqaaaqaaiab=T7aSnaaBaaaleaacqWGSbaBcqWGPbqAaeqaaOGaeyypa0JaeeiDaqNaeeiAaGMaeeyzauMaeeiiaaIaemyAaK2aaWbaaSqabeaacqWG0baDcqWGObaAaaGccqqGLbqzcqqGPbqAcqqGNbWzcqqGLbqzcqqGUbGBcqqG2bGDcqqGHbqycqqGSbaBcqqG1bqDcqqGLbqzcqqGGaaicqqGPbqAcqqGUbGBcqqGGaaicqWGSbaBdaahaaWcbeqaaiabdsha0jabdIgaObaakiabbogaJjabbYgaSjabbwha1jabbohaZjabbsha0jabbwgaLjabbkhaYbaaaaa@C719@

In this paper, eigenvalues are calculated from the within-cluster covariance matrix and assumed to be sorted in decreasing order so that the first eigenvalue corresponds to the largest eigenvalue, the second eigenvalue corresponds to the second largest eigenvalue and so on. From a dimension reduction perspective, the principal components of each resulting cluster lie in the directions of the axes of a constant density multi-dimensional ellipsoid [24]. If the relative magnitude of the first eigenvalue is large then the corresponding cluster is closer to linear in shape. Recently, Moller-Levet *et al*. used the square root of the second eigenvalue as an overall clustering quality index for microarray data [13]. In the spirit of Moller-Levet, the ratio of the normalized eigenvalue to the total number of clusters is used as an evaluation measure.

**Figure 3:**
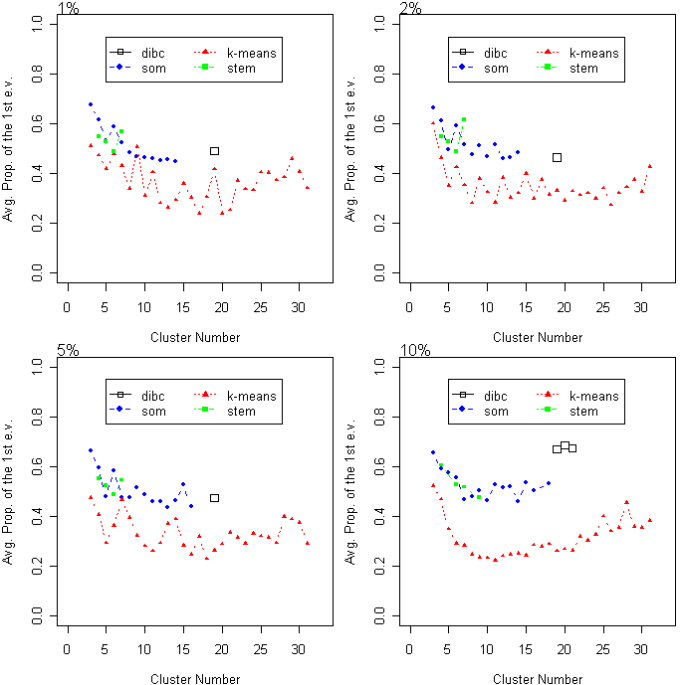
**APF of the simulated data**. The average proportion of the first eigenvalue (APF) is plotted as a function of cluster number. Higher APF values indicate that clusters are closer to a linear- shape. Three algorithms were compared at four different noise levels.

With the APF measure, DIB-C had the largest value in the neighborhood of the true cluster number 19 under 1, 2, and 10% noise (Figure 3). The dots of DIB-C appeared close to the true cluster number 19 and stayed only in the neighborhood of 19 with its APF values being the highest. Based on this result, we argue that DIB-C produces mostly linear-shaped clusters because it has the largest proportion of the first eigenvalue of each cluster covariance.

**Figure 4:**
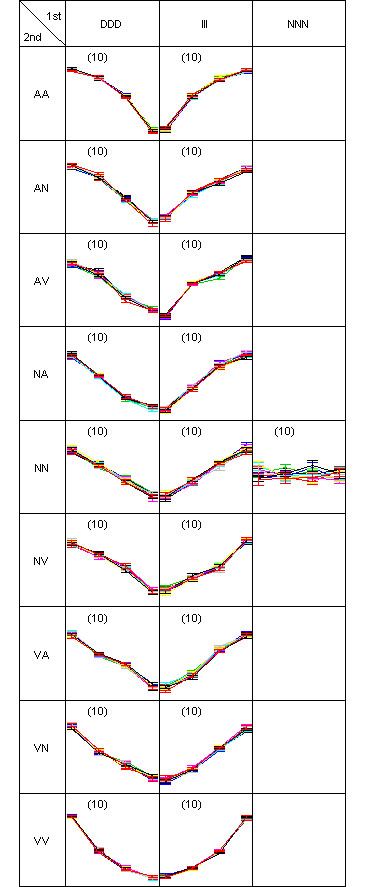
**pattern map of simulation scheme**. Two-dimensional pattern map showing simulated data for 190 genes at four time-points. Nineteen clusters are predefined. Each cluster has ten genes. Every gene has eight replicates. The symbols are I: increase, D: decrease, N: no-change, A: concave and V: convex.

**Figure 5:**
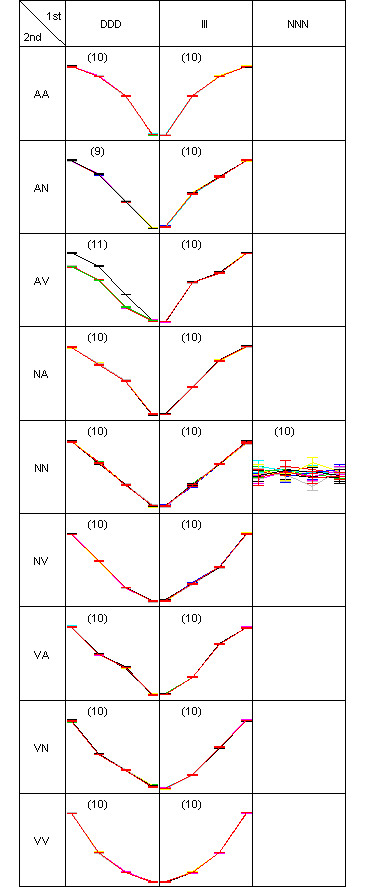
**pattern map of the simulated data**. Two-dimensional pattern map of the clustering test data., in which 190 genes are partitioned into 19 clusters. There was only one misclassified gene in the pattern (DDD, AV), so ARI was 1.

A two-dimensional pattern map (as described in the Methods section) is shown to explain how the simulation data was generated (Figure 4). Each gene is assigned to the true cluster where three first-order difference pattern on the columns are further partitioned into nine second-order patterns on the rows. In each gene, error bars are drawn around the mean for each time-points. Ten member genes constitute a cluster with a total of 19 true clusters. Then the clustering result of DIB-C is shown in Figure 5 to compare with the truth (Figure 4). The result is similar to the true answer since there was only one mis-clustered gene in the cluster (DDD, AV) whose cluster size is 11. Actual membership of this gene is (DDD, AN) whose cluster size is 9.

**Figure 6:**
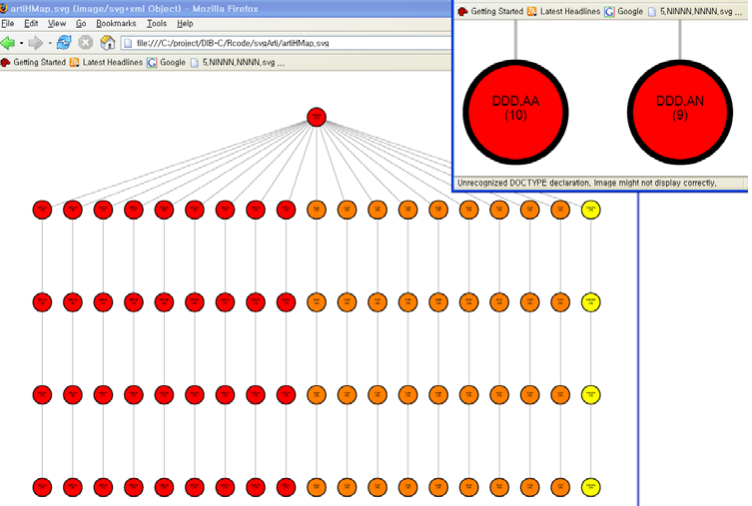
**hierarchical layer of the simulated data**. Clustering results of simulated data are drawn as a hierarchical graph. Each level is a clustering result from each threshold value. Since every clustering result is the same for all levels, except level 0, DAG is drawn only up to level 5. Each node represents a symbolic pattern. The number of members in each cluster is written in parentheses. An interactive figure in SVG format is available on the supplement page [27].

The hierarchical layers of the simulated data are shown in Figure 6; the four smallest thresholds are shown in this figure because the complete figure is so complex. Every threshold level uniformly, and correctly, exhibited 19 clusters. After level 1, there is no further repartitioning of a cluster and the three first differences are observed as three corresponding colors in each level.

### Real data

For real data, gene set enrichment using Gene Ontology (GO) annotation was used instead of ARI (in the simulated data) since true cluster membership is not available. GO is a structured, controlled vocabulary for describing the roles of genes and gene products [25]. Following the work of Gibbons *et al*., the molecular function aspect was used out of the three aspects of GO. After mapping the third-level GO ID to our pancreas genes, a contingency table of 2,179 genes by 13,505 GO IDs was created. Then the total mutual information *MI*_*real *_between the cluster result and all the GO IDs were computed. Next, *MI*_*random *_for a clustering result was obtained after random swapping of genes in the original clusters. This procedure was repeated 3,000 times to get corresponding *MI*_*random*_s. Then, we subtracted the mean of *MI*_*random*_s from *MI*_*real *_and divided it by the standard deviation of *MI*_*random*_s. This is a Z-score interpreted as a standardized distance between the *MI *value obtained from clustering after centering and scaling based on those *MI *values obtained by random assignments of genes to clusters. The higher the Z-score, the better one's clustering result because it indicates the observed clustering result is further away from the distribution of the random clustering results [26].

**Figure 7:**
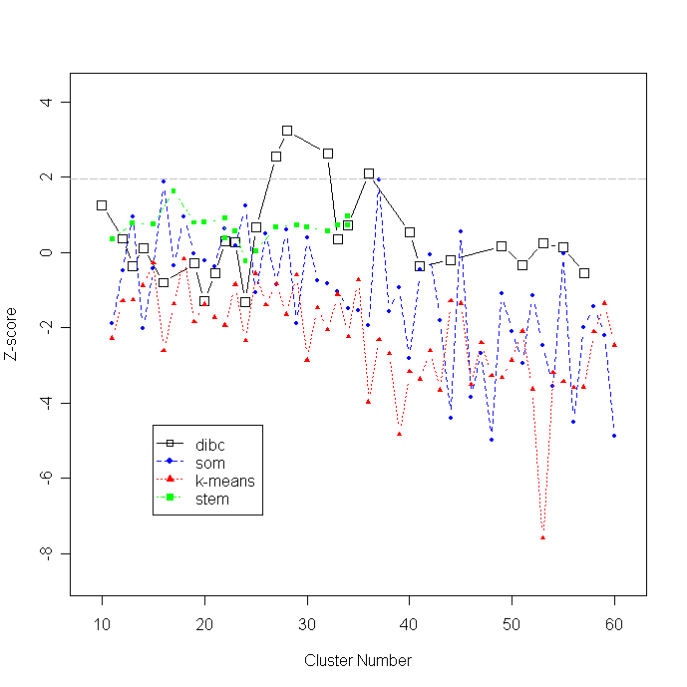
**Z-scores of pancreas data**. Mutual information between a clustering result and GO annotation is plotted using the cluster number. Higher Z-scores indicate better clustering results based on external knowledge, GO. The optimal cluster number is 28 where the maximum Z-score, 3.247, is achieved.

Z-score for the pancreas data is shown in Figure 7. Overall trend of the *Z*-scores for the mutual information between clustering results and significant GO annotation for the real data decreases with an increase in cluster size, as noted by Gibbons *et al*.. When significant *Z*-scores were considered (*i.e. Z*-scores higher than 97.5% normal-quantile, 1.96), DIB-C gave higher and more stable Z-score values (Figure 7), achieving more significant and insightful clusters. DIB-C had the largest *Z*-score (Z = 3.247) at 28 clusters. This was obtained from the first- and the second-order thresholds pair of p-value cutoffs (9 × 10^-4^, 3 × 10^-4^) for significant differences.

**Figure 8:**
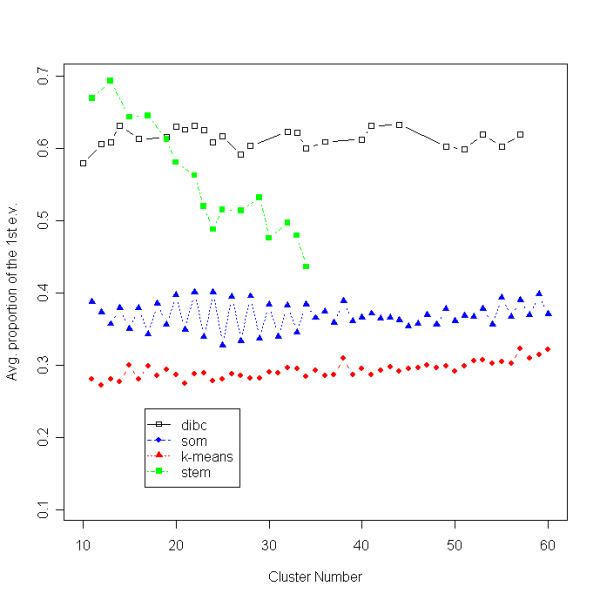
**APF of pancreas data**. The average proportion of the first eigenvalue (APF) is plotted as a function of cluster number.

With the APF measure, both DIB-C and STEM outperformed SOM and *K*-means (Figure 8). DIB-C gave the largest APF values across all cluster numbers larger than 19. STEM had the largest APF values when the cluster number was smaller than 19, but the differences in APF values between STEM and DIB-C were small. While DIB-C showed stable APF values, STEM's values decreased as the cluster number increased. Overall, DIB-C had the largest (or nearly the largest) average magnitude of the first eigenvalue in each cluster.

**Figure 9:**
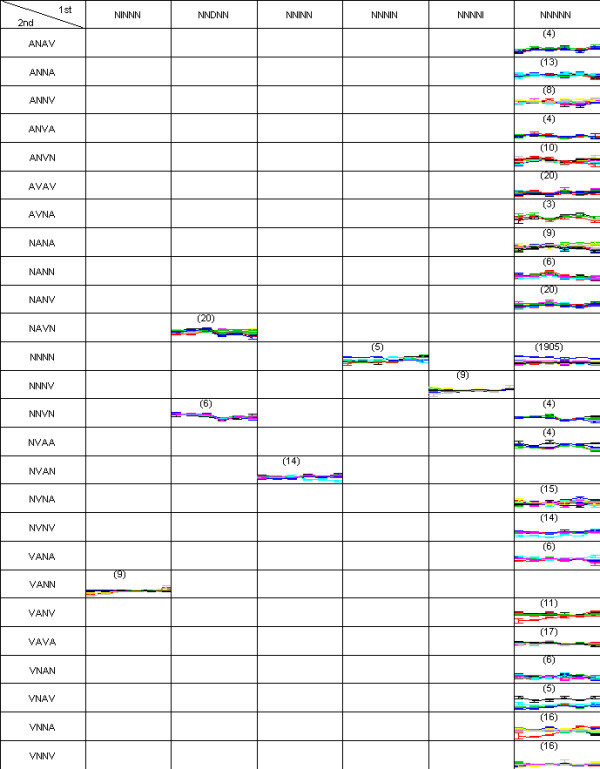
**hierarchical layer of pancreas data**. Hierarchal structure of clustering results are drawn as a from the pancreas data. Three layers (or clustering results) are attached to the root produced from the corresponding cutoff pairs of (1 × 10^-5^, 2 × 10^-5^), (6 × 10^-4^, 1 × 10^-5^), and (9 × 10^-4^, 3 × 10^-3^). An interactive figure in SVG format is available on the supplement page [27].

For the pancreas dataset, three representative threshold levels, including the 'optimal' result with 28 clusters (Z-score = 3.247), are applied to construct the corresponding three hierarchical layers (Figure 9). Levels 1 and 2 are included as ancestor layers of the optimal layer. Level 1 had a *Z*-score of 1.258 at cluster number 10 with threshold pair (1 × 10^-5^, 2 × 10^-5^); Level 2 had a *Z*-score of 2.557 at cluster number 28 with threshold pair (7 × 10^-4^, 1 × 10^-5^). An interactive version of this hierarchical layer can be found at the supplementary webpage [27].

**Figure 10:**
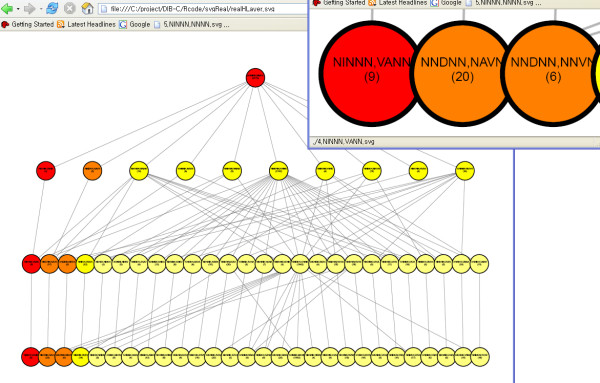
**pattern map of pancreas data**. Two-dimensional pattern map of the pancreas gene expression data. There were 2,179 genes and six time-points. T1 had four replicates and the other time-points had six. A cutoff value 0.003 was used to identify significant differences.

The optimal clustering result from the last hierarchical layer is reconstructed as a two-dimensional pattern map for the pancreas data (Figure 10). DIB-C partitioned 2,179 probes of pancreas data into 28 clusters. The pattern map had six first-differences and 25 second-differences. As expected, a huge cluster (1,905 genes, 87.4%) of the null pattern ((*N*, *N*, *N*, *N*, *N*), (*N*, *N*, *N*, *N*)) was found.

## Discussion and conclusions

DIB-C is a novel clustering algorithm based on the first- and second-order differences of a gene expression matrix. Our algorithm has several advantages over previous clustering algorithms for short time-course data with replicates. First, DIB-C generates interpretable clusters through discretization. Instead of producing many unlabeled partitions, DIB-C offers self-explanatory clusters. The resulting pattern map visualizes using both horizontal (the first-order difference) and vertical (the second-order difference) structures. Each cluster has a label composed of symbols indicating increases or decreases, which have intuitive biological interpretations. Second, our algorithm deals with the rate of change: convex and concave categories are incorporated into the definition of the symbolic pattern. Hence, we can discriminate genes into further subgroups. Third, the identification power is increased by using both the mean and variance of replicates. Conventional algorithms blindly use averaged summary data from replicates. In this way, two average values with different variances are treated equally, thereby decreasing the sensitivity to non-random patterns. Fourth, temporal order is incorporated into the algorithm. Column-wise shuffling (*i.e*., re-ordering time-points) of input data would give a different output, which is not the case for *K *-means or SOM because they do not consider the order of input data points. Fifth, DIB-C requires no prior knowledge of representative genes. Even after the appropriate clustering algorithm is chosen, deciding the optimal number of clusters is very important. DIB-C overcomes this problem by exhaustive space searching in an efficient way. Also, DIB-C offers informative visualization. Clusters are arranged so that closely related patterns are gathered together. Such a meta-structure approach is often needed in developmental and cancer biology.

When a cluster has few members, the APF value tends to get large since most eigenvalues of its within-cluster covariance matrix could be zeros. For this potential bias of APF measure, we have assigned equally 10 members to each 19 cluster in the simulated data. APF values had a tendency to increase as the cluster number decreased (Figure 3) in the clustering algorithms other than DIB-C. But our algorithm produced the highest APF value at the true cluster number 19 and only around this number. This result tells us that there might be a small bias in favor of the smaller number of the cluster, but not big enough to mask the performance of DIB-C over the other algorithms. For real data, DIB-C produced a huge null cluster having the majority of genes and a small number of clusters having a few members. However, note that in practice, a filtering step often precedes clustering methods based on the assumption that most genes are not expressed significantly under the conditions of microarray experiments. Hence the final clustering results are affected by the choice of filtering criteria, making the optimal partitioning (of genes) problem more complex and potentially biased in other direction. In contrast, DIB-C performs filtering and clustering simultaneously because the all-null-pattern ((*N*, *N*, ...), (*N*, *N*, ...)) is just another symbolic pattern in our algorithm. By excluding this null cluster, simultaneous filtering and clustering can be performed, unlike in other clustering algorithms. Tseng *et al*. [28] also criticized that current clustering algorithms are forced to assign every gene to a cluster. Many genes are irrelevant to biological pathways or conditions under screening and so the main interest of investigators lies in identifying the most informative, clusters of small sizes. With this in mind, we consider that the best clustering algorithm should produce a huge cluster of "irrelevant genes" (which corresponds to our null pattern) and a small number of clusters having a few members and this is exactly what DIB-C does.

Our algorithm is based on conceptual discretizations, such as increasing, decreasing, remaining flat, and convexity or concavity, that are used as basic building blocks to define a pattern or cluster that shares a pattern with itself. This makes each cluster meaningful and interpretable. Although discretiztion may cause some loss of information, what investigators expect in gene expression data with time course may be such simple statements such as: Which genes express more rapidly with an increase of time? Which genes express early and late but remain flat in the middle? Simply finding patterns and clusters may not be sufficient for the biologists, but it is our belief that we need more effort to relate, even at the cost of loosing some information, computational analysis results with biological phenomena.

For the exhaustive search, DIB-C iterates |*T*|^2 ^× (2*p *- 3) times where |*T*| is the number of threshold values in the Methods section and *p *is the number of time-points. In practice, the investigator would not need to use as many threshold values as in this study since there are multiple testing issues. Common choice of T would be *T *= {1 × 10^-5^, 2 × 10^-5^, ..., 9 × 10^-5^, ..., 1 × 10^-4^, ..., 9 × 10^-4^} which has a length 18. The runtime of our algorithm increases exponentially with the number of time-points. However, most publicly available gene expression datasets have only a small number of time-points. According to the survey by Ernst *et. al*. [12], most datasets involved only 2~8 time-points. If the number of time-points is very high, conventional time-series techniques can instead be used; DIB-C is designed for situations where this is not the case.

DIB-C can be generalized for use on other types of ordinal data, including stress-response or drug-treatment data, although the example datasets presented here focus on time-course data. In the future, we plan to extend DIB-C to two-factor designs whose orders are in two directions. For example, the analysis of drug-induced gene expression has both time-order and treatment dose-order. We could apply DIB-C after redefining the first-and second-order differences in order to take this account.

## Methods

### Data

We synthesized a test set of expression data for 190 genes at four time-points and with eight replicates. The range of log-ratio values was (-4, 4). Based on 19 template genes representing 19 clusters, we generated ten member genes for each cluster with uniform noise *Unif *(-0.01, 0.01), obtaining each such gene using a 190 by 4 condition matrix. For replicates, we added normal noise using *N *(0, *σ*^2^) where *σ *is taken from {0.04, 0.08, 0.2, 0.4}, so the matrix was extended to 190 by 32.

We also validated our algorithm with a real dataset involving pancreas gene expression in developing mice [21]. There were 3,840 genes and six time-points (embryonic day E14.5, E16.5, E18.5, birth, postnatal day P7, and adulthood). Six replicates were performed for each time-point from E16.5 through adulthood. E14.5 had only four replicates due to the low amount of mRNA [21]. We extracted 2,179 unique probes from the original 3,840: First, we filtered out genes if they were either unidentified or could not be associated with a GO ID. Then, we averaged redundant genes. For preprocessing, scaled print-tip group *lowess *(locally weighted scatter plot smoothing) was performed to remove these spatial effects to enable fair comparison across time-points [29].

### Algorithm

An outline of the algorithm is as following. For each gene, a (moderated) t-statistic is obtained for two adjacent time-points. If the initial number of time-points was *p*, we should have a vector of (*p *- 1) t-statistics for each gene. Then each t-statistic is categorized into one of three symbols *I *(Increase), *D *(Decrease) or *N *(No change) depending on the t-distribution and the predefined cutoff. This constitutes the first-order symbolic pattern vector of length (*p *- 1). Next, the difference of two adjacent t-statistics is calculated. Each difference is discretized into one of three symbols *V *(conVex), *A *(concAve) or *N *(No change) depending on the (empirical) distribution and the cutoff. These symbols constitute the second-order symbolic pattern vector of length (*p *- 2). Last a symbolic pattern of vector of length (*p *- 1) + (*p *- 2) = (2*p *- 3) is defined. Once the symbolic pattern is obtained, the gene is automatically assigned to this pattern and the above procedures are repeated for each gene independently.

There are two inputs for the proposed algorithm: the gene expression data and the experimental design matrix.

Gene expression data *Y *= {*y*_*gjk*_} where,

g=1,...,ngenej=1,...,ptime-pointk=1,...,mjreplicate for each (g,j)
 MathType@MTEF@5@5@+=feaafiart1ev1aaatCvAUfKttLearuWrP9MDH5MBPbIqV92AaeXatLxBI9gBaebbnrfifHhDYfgasaacH8akY=wiFfYdH8Gipec8Eeeu0xXdbba9frFj0=OqFfea0dXdd9vqai=hGuQ8kuc9pgc9s8qqaq=dirpe0xb9q8qiLsFr0=vr0=vr0dc8meaabaqaciaacaGaaeqabaqabeGadaaakeaafaqaaeWacaaabaGaem4zaCMaeyypa0JaeGymaeJaeiilaWIaeiOla4IaeiOla4IaeiOla4IaeiilaWIaemOBa4gabaGaee4zaCMaeeyzauMaeeOBa4MaeeyzaugabaGaemOAaOMaeyypa0JaeGymaeJaeiilaWIaeiOla4IaeiOla4IaeiOla4IaeiilaWIaemiCaahabaGaeeiDaqNaeeyAaKMaeeyBa0MaeeyzauMaeeyla0IaeeiCaaNaee4Ba8MaeeyAaKMaeeOBa4MaeeiDaqhabaGaem4AaSMaeyypa0JaeGymaeJaeiilaWIaeiOla4IaeiOla4IaeiOla4IaeiilaWIaemyBa02aaSbaaSqaaiabdQgaQbqabaaakeaacqqGYbGCcqqGLbqzcqqGWbaCcqqGSbaBcqqGPbqAcqqGJbWycqqGHbqycqqG0baDcqqGLbqzcqqGGaaicqqGMbGzcqqGVbWBcqqGYbGCcqqGGaaicqqGLbqzcqqGHbqycqqGJbWycqqGObaAcqqGGaaicqGGOaakcqqGNbWzcqGGSaalcqqGQbGAcqGGPaqkaaaaaa@7990@

Experimental design matrix *Q *= {*q*_*jl*_}_*n *× 2 _where,

j=1,2,...,p;l=1,2qj1=jth time-point'level name'qj2=mj'sample size'
 MathType@MTEF@5@5@+=feaafiart1ev1aaatCvAUfKttLearuWrP9MDH5MBPbIqV92AaeXatLxBI9gBaebbnrfifHhDYfgasaacH8akY=wiFfYdH8Gipec8Eeeu0xXdbba9frFj0=OqFfea0dXdd9vqai=hGuQ8kuc9pgc9s8qqaq=dirpe0xb9q8qiLsFr0=vr0=vr0dc8meaabaqaciaacaGaaeqabaqabeGadaaakeaafaqaaeWacaaabaGaemOAaOMaeyypa0JaeGymaeJaeiilaWIaeGOmaiJaeiilaWIaeiOla4IaeiOla4IaeiOla4IaeiilaWIaemiCaaNaei4oaSdabaGaemiBaWMaeyypa0JaeGymaeJaeiilaWIaeGOmaidabaGaemyCae3aaSbaaSqaaiabdQgaQjabigdaXaqabaGccqGH9aqpcqWGQbGAdaahaaWcbeqaaiabdsha0jabdIgaObaakiabbccaGiabbsha0jabbMgaPjabb2gaTjabbwgaLjabb2caTiabbchaWjabb+gaVjabbMgaPjabb6gaUjabbsha0bqaaiabcEcaNiabbYgaSjabbwgaLjabbAha2jabbwgaLjabbYgaSjabbccaGiabb6gaUjabbggaHjabb2gaTjabbwgaLjabcEcaNaqaaiabdghaXnaaBaaaleaacqWGQbGAcqaIYaGmaeqaaOGaeyypa0JaemyBa02aaSbaaSqaaiabdQgaQbqabaaakeaacqGGNaWjcqqGZbWCcqqGHbqycqqGTbqBcqqGWbaCcqqGSbaBcqqGLbqzcqqGGaaicqqGZbWCcqqGPbqAcqqG6bGEcqqGLbqzcqGGNaWjaaaaaa@7C0D@

#### Step 1: The first-order difference

The first-order difference matrix Y(1)={ygj(1)}n×(p−1)
 MathType@MTEF@5@5@+=feaafiart1ev1aaatCvAUfKttLearuWrP9MDH5MBPbIqV92AaeXatLxBI9gBaebbnrfifHhDYfgasaacH8akY=wiFfYdH8Gipec8Eeeu0xXdbba9frFj0=OqFfea0dXdd9vqai=hGuQ8kuc9pgc9s8qqaq=dirpe0xb9q8qiLsFr0=vr0=vr0dc8meaabaqaciaacaGaaeqabaqabeGadaaakeaacqWGzbqwdaahaaWcbeqaaiabcIcaOiabigdaXiabcMcaPaaakiabg2da9iabcUha7jabdMha5naaDaaaleaacqWGNbWzcqWGQbGAaeaacqGGOaakcqaIXaqmcqGGPaqkaaGccqGG9bqFdaWgaaWcbaGaemOBa4Maey41aqRaeiikaGIaemiCaaNaeyOeI0IaeGymaeJaeiykaKcabeaaaaa@446E@ is derived from Y where

ygj(1)=β^gjs˜gvgjmoderated t-statisticβ^gjthe mean difference between two groups;jth and (j+1)th time-pointss˜gposterior mean of sample variance for gene gvgjsample variance for two groups of gene g;jth and (j+1)th time-points
 MathType@MTEF@5@5@+=feaafiart1ev1aaatCvAUfKttLearuWrP9MDH5MBPbIqV92AaeXatLxBI9gBaebbnrfifHhDYfgasaacH8akY=wiFfYdH8Gipec8Eeeu0xXdbba9frFj0=OqFfea0dXdd9vqai=hGuQ8kuc9pgc9s8qqaq=dirpe0xb9q8qiLsFr0=vr0=vr0dc8meaabaqaciaacaGaaeqabaqabeGadaaakeaafaqaaeabcaaaaeaacqWG5bqEdaqhaaWcbaGaem4zaCMaemOAaOgabaGaeiikaGIaeGymaeJaeiykaKcaaOGaeyypa0ZaaSaaaeaaiiGacuWFYoGygaqcamaaBaaaleaacqWGNbWzcqWGQbGAaeqaaaGcbaGafm4CamNbaGaadaWgaaWcbaGaem4zaCgabeaakmaakaaabaGaemODay3aaSbaaSqaaiabdEgaNjabdQgaQbqabaaabeaaaaaakeaacqqGTbqBcqqGVbWBcqqGKbazcqqGLbqzcqqGYbGCcqqGHbqycqqG0baDcqqGLbqzcqqGKbazcqqGGaaicqqG0baDcqqGTaqlcqqGZbWCcqqG0baDcqqGHbqycqqG0baDcqqGPbqAcqqGZbWCcqqG0baDcqqGPbqAcqqGJbWyaeaacuWFYoGygaqcamaaBaaaleaacqWGNbWzcqWGQbGAaeqaaaGcbaGaeeiDaqNaeeiAaGMaeeyzauMaeeiiaaIaeeyBa0MaeeyzauMaeeyyaeMaeeOBa4MaeeiiaaIaeeizaqMaeeyAaKMaeeOzayMaeeOzayMaeeyzauMaeeOCaiNaeeyzauMaeeOBa4Maee4yamMaeeyzauMaeeiiaaIaeeOyaiMaeeyzauMaeeiDaqNaee4DaCNaeeyzauMaeeyzauMaeeOBa4MaeeiiaaIaeeiDaqNaee4DaCNaee4Ba8MaeeiiaaIaee4zaCMaeeOCaiNaee4Ba8MaeeyDauNaeeiCaaNaee4CamNaei4oaSJaemOAaO2aaWbaaSqabeaacqWG0baDcqWGObaAaaGccqqGGaaicqqGHbqycqqGUbGBcqqGKbazcqqGGaaicqGGOaakcqWGQbGAcqGHRaWkcqaIXaqmcqGGPaqkdaahaaWcbeqaaiabdsha0jabdIgaObaakiabbccaGiabbsha0jabbMgaPjabb2gaTjabbwgaLjabb2caTiabbchaWjabb+gaVjabbMgaPjabb6gaUjabbsha0jabbohaZbqaaiqbdohaZzaaiaWaaSbaaSqaaiabdEgaNbqabaaakeaacqqGWbaCcqqGVbWBcqqGZbWCcqqG0baDcqqGLbqzcqqGYbGCcqqGPbqAcqqGVbWBcqqGYbGCcqqGGaaicqqGTbqBcqqGLbqzcqqGHbqycqqGUbGBcqqGGaaicqqGVbWBcqqGMbGzcqqGGaaicqqGZbWCcqqGHbqycqqGTbqBcqqGWbaCcqqGSbaBcqqGLbqzcqqGGaaicqqG2bGDcqqGHbqycqqGYbGCcqqGPbqAcqqGHbqycqqGUbGBcqqGJbWycqqGLbqzcqqGGaaicqqGMbGzcqqGVbWBcqqGYbGCcqqGGaaicqqGNbWzcqqGLbqzcqqGUbGBcqqGLbqzcqqGGaaicqWGNbWzaeaacqWG2bGDdaWgaaWcbaGaem4zaCMaemOAaOgabeaaaOqaaiabbohaZjabbggaHjabb2gaTjabbchaWjabbYgaSjabbwgaLjabbccaGiabbAha2jabbggaHjabbkhaYjabbMgaPjabbggaHjabb6gaUjabbogaJjabbwgaLjabbccaGiabbAgaMjabb+gaVjabbkhaYjabbccaGiabbsha0jabbEha3jabb+gaVjabbccaGiabbEgaNjabbkhaYjabb+gaVjabbwha1jabbchaWjabbohaZjabbccaGiabb+gaVjabbAgaMjabbccaGiabbEgaNjabbwgaLjabb6gaUjabbwgaLjabbccaGiabdEgaNjabcUda7iabdQgaQnaaCaaaleqabaGaemiDaqNaemiAaGgaaOGaeeiiaaIaeeyyaeMaeeOBa4MaeeizaqMaeeiiaaIaeiikaGIaemOAaOMaey4kaSIaeGymaeJaeiykaKYaaWbaaSqabeaacqWG0baDcqWGObaAaaGccqqGGaaicqqG0baDcqqGPbqAcqqGTbqBcqqGLbqzcqqGTaqlcqqGWbaCcqqGVbWBcqqGPbqAcqqGUbGBcqqG0baDcqqGZbWCaaaaaa@48B8@

ygj(1)
 MathType@MTEF@5@5@+=feaafiart1ev1aaatCvAUfKttLearuWrP9MDH5MBPbIqV92AaeXatLxBI9gBaebbnrfifHhDYfgasaacH8akY=wiFfYdH8Gipec8Eeeu0xXdbba9frFj0=OqFfea0dXdd9vqai=hGuQ8kuc9pgc9s8qqaq=dirpe0xb9q8qiLsFr0=vr0=vr0dc8meaabaqaciaacaGaaeqabaqabeGadaaakeaacqWG5bqEdaqhaaWcbaGaem4zaCMaemOAaOgabaGaeiikaGIaeGymaeJaeiykaKcaaaaa@33AA@ is a two-sample (moderated) t-statistic between adjacent *j*^*th *^and (*j *+ 1)^*th *^time-point groups with *g *respective sample sizes of nRj
 MathType@MTEF@5@5@+=feaafiart1ev1aaatCvAUfKttLearuWrP9MDH5MBPbIqV92AaeXatLxBI9gBaebbnrfifHhDYfgasaacH8akY=wiFfYdH8Gipec8Eeeu0xXdbba9frFj0=OqFfea0dXdd9vqai=hGuQ8kuc9pgc9s8qqaq=dirpe0xb9q8qiLsFr0=vr0=vr0dc8meaabaqaciaacaGaaeqabaqabeGadaaakeaacqWGUbGBdaWgaaWcbaGaemOuai1aaSbaaWqaaiabdQgaQbqabaaaleqaaaaa@30FF@ and nRj+1
 MathType@MTEF@5@5@+=feaafiart1ev1aaatCvAUfKttLearuWrP9MDH5MBPbIqV92AaeXatLxBI9gBaebbnrfifHhDYfgasaacH8akY=wiFfYdH8Gipec8Eeeu0xXdbba9frFj0=OqFfea0dXdd9vqai=hGuQ8kuc9pgc9s8qqaq=dirpe0xb9q8qiLsFr0=vr0=vr0dc8meaabaqaciaacaGaaeqabaqabeGadaaakeaacqWGUbGBdaWgaaWcbaGaemOuai1aaSbaaWqaaiabdQgaQjabgUcaRiabigdaXaqabaaaleqaaaaa@32D1@. This empirical Bayes method was proposed by Smyth *et al*. [15] and it reduces the observed variances towards a pooled estimate, thereby providing a more stable inference when the number of replicates is small.

#### Step 2: Symbolic pattern matrix *F*

*Y*^(1) ^is categorized into three symbols *I *(Increase), *D *(Decrease) or *N *(No change) to get the pattern matrix *F *= {*f*_*gj*_}_*n *× (*p *- 1) _based on the critical value from the t-distribution. This step is a usual two-sample t-test.

fgj=I,if ygj(1)>T(1−α/2;dfgj)=D,if ygj(1)<T(1−α/2;dfgj)=N,otherwise
 MathType@MTEF@5@5@+=feaafiart1ev1aaatCvAUfKttLearuWrP9MDH5MBPbIqV92AaeXatLxBI9gBaebbnrfifHhDYfgasaacH8akY=wiFfYdH8Gipec8Eeeu0xXdbba9frFj0=OqFfea0dXdd9vqai=hGuQ8kuc9pgc9s8qqaq=dirpe0xb9q8qiLsFr0=vr0=vr0dc8meaabaqaciaacaGaaeqabaqabeGadaaakeaafaqaaeWaeaaaaeaacqWGMbGzdaWgaaWcbaGaem4zaCMaemOAaOgabeaaaOqaaiabg2da9aqaaiabdMeajjabcYcaSaqaaiabbMgaPjabbAgaMjabbccaGiabdMha5naaDaaaleaacqWGNbWzcqWGQbGAaeaacqGGOaakcqaIXaqmcqGGPaqkaaGccqGH+aGpcqWGubavcqGGOaakcqaIXaqmcqGHsisliiGacqWFXoqycqGGVaWlcqaIYaGmcqGG7aWocqWGKbazcqWGMbGzdaWgaaWcbaGaem4zaCMaemOAaOgabeaakiabcMcaPaqaaaqaaiabg2da9aqaaiabdseaejabcYcaSaqaaiabbMgaPjabbAgaMjabbccaGiabdMha5naaDaaaleaacqWGNbWzcqWGQbGAaeaacqGGOaakcqaIXaqmcqGGPaqkaaGccqGH8aapcqWGubavcqGGOaakcqaIXaqmcqGHsislcqWFXoqycqGGVaWlcqaIYaGmcqGG7aWocqWGKbazcqWGMbGzdaWgaaWcbaGaem4zaCMaemOAaOgabeaakiabcMcaPaqaaaqaaiabg2da9aqaaiabd6eaojabcYcaSaqaaiabb+gaVjabbsha0jabbIgaOjabbwgaLjabbkhaYjabbEha3jabbMgaPjabbohaZjabbwgaLbaaaaa@7B09@

where *g *= 1,2,..., *n*; *j *= 1,2,..., *p*-1

*df*_*gj *_is the empirically estimated degree of freedom by [15]

#### Step 3: The second-order difference

The second-order difference matrix Y(2)={ygj(2)}n×(p−2)
 MathType@MTEF@5@5@+=feaafiart1ev1aaatCvAUfKttLearuWrP9MDH5MBPbIqV92AaeXatLxBI9gBaebbnrfifHhDYfgasaacH8akY=wiFfYdH8Gipec8Eeeu0xXdbba9frFj0=OqFfea0dXdd9vqai=hGuQ8kuc9pgc9s8qqaq=dirpe0xb9q8qiLsFr0=vr0=vr0dc8meaabaqaciaacaGaaeqabaqabeGadaaakeaacqWGzbqwdaahaaWcbeqaaiabcIcaOiabikdaYiabcMcaPaaakiabg2da9iabcUha7jabdMha5naaDaaaleaacqWGNbWzcqWGQbGAaeaacqGGOaakcqaIYaGmcqGGPaqkaaGccqGG9bqFdaWgaaWcbaGaemOBa4Maey41aqRaeiikaGIaemiCaaNaeyOeI0IaeGOmaiJaeiykaKcabeaaaaa@4474@ is obtained by subtracting the first-order differences.

ygj(2)=yg(j+1)(1)−ygj(1)g=1,2,...,n;j=1,2,...,p−2
 MathType@MTEF@5@5@+=feaafiart1ev1aaatCvAUfKttLearuWrP9MDH5MBPbIqV92AaeXatLxBI9gBaebbnrfifHhDYfgasaacH8akY=wiFfYdH8Gipec8Eeeu0xXdbba9frFj0=OqFfea0dXdd9vqai=hGuQ8kuc9pgc9s8qqaq=dirpe0xb9q8qiLsFr0=vr0=vr0dc8meaabaqaciaacaGaaeqabaqabeGadaaakeaafaqaaeGacaaabaGaemyEaK3aa0baaSqaaiabdEgaNjabdQgaQbqaaiabcIcaOiabikdaYiabcMcaPaaakiabg2da9iabdMha5naaDaaaleaacqWGNbWzcqGGOaakcqWGQbGAcqGHRaWkcqaIXaqmcqGGPaqkaeaacqGGOaakcqaIXaqmcqGGPaqkaaGccqGHsislcqWG5bqEdaqhaaWcbaGaem4zaCMaemOAaOgabaGaeiikaGIaeGymaeJaeiykaKcaaaGcbaaabaGaem4zaCMaeyypa0JaeGymaeJaeiilaWIaeGOmaiJaeiilaWIaeiOla4IaeiOla4IaeiOla4IaeiilaWIaemOBa4Maei4oaSdabaGaemOAaOMaeyypa0JaeGymaeJaeiilaWIaeGOmaiJaeiilaWIaeiOla4IaeiOla4IaeiOla4IaeiilaWIaemiCaaNaeyOeI0IaeGOmaidaaaaa@6013@

#### Step 4: Symbolic pattern matrix *S*

*Y*^(2) ^is discretized into three symbols *V *(conVex), *A *(concAve), or *N *(No change) to get the symbolic pattern matrix *S *= {*f*_*gj*_}_*n *× (*p*-2)_. Here, the critical values were set using the difference of t-distributions, say *T*', empirically. To get the quantiles of *T*', two random samples of size 10,000 were generated from the t-distribution with degrees of freedom *m*_*j *_- 1 and *m*_(*j*+1) _- 1, respectively. Then, the *α*^*th *^quantile values from the difference of the two samples were saved. This procedure was repeated 1,000 times. Finally, the median value of the 1,000 quantile values was chosen as our final critical value.

sgj=V,if ygj(2)>(1−α)thquantile of T′=A,if ygj(2)<αthquantile of T′=N,otherwiseg=1,2,...,n;j=1,2,...,p−2
 MathType@MTEF@5@5@+=feaafiart1ev1aaatCvAUfKttLearuWrP9MDH5MBPbIqV92AaeXatLxBI9gBaebbnrfifHhDYfgasaacH8akY=wiFfYdH8Gipec8Eeeu0xXdbba9frFj0=OqFfea0dXdd9vqai=hGuQ8kuc9pgc9s8qqaq=dirpe0xb9q8qiLsFr0=vr0=vr0dc8meaabaqaciaacaGaaeqabaqabeGadaaakeaafaqaaeabeaaaaaqaaiabdohaZnaaBaaaleaacqWGNbWzcqWGQbGAaeqaaaGcbaGaeyypa0dabaGaemOvayLaeiilaWcabaGaeeyAaKMaeeOzayMaeeiiaaIaemyEaK3aa0baaSqaaiabdEgaNjabdQgaQbqaaiabcIcaOiabikdaYiabcMcaPaaakiabg6da+iabcIcaOiabigdaXiabgkHiTGGaciab=f7aHjabcMcaPmaaCaaaleqabaGaemiDaqNaemiAaGgaaOGaeeyCaeNaeeyDauNaeeyyaeMaeeOBa4MaeeiDaqNaeeyAaKMaeeiBaWMaeeyzauMaeeiiaaIaee4Ba8MaeeOzayMaeeiiaaIafmivaqLbauaaaeaaaeaacqGH9aqpaeaacqWGbbqqcqGGSaalaeaacqqGPbqAcqqGMbGzcqqGGaaicqWG5bqEdaqhaaWcbaGaem4zaCMaemOAaOgabaGaeiikaGIaeGOmaiJaeiykaKcaaOGaeyipaWJae8xSde2aaWbaaSqabeaacqWG0baDcqWGObaAaaGccqqGXbqCcqqG1bqDcqqGHbqycqqGUbGBcqqG0baDcqqGPbqAcqqGSbaBcqqGLbqzcqqGGaaicqqGVbWBcqqGMbGzcqqGGaaicuWGubavgaqbaaqaaaqaaiabg2da9aqaaiabd6eaojabcYcaSaqaaiabb+gaVjabbsha0jabbIgaOjabbwgaLjabbkhaYjabbEha3jabbMgaPjabbohaZjabbwgaLbqaaaqaaaqaaaqaauaabeqabiaaaeaacqWGNbWzcqGH9aqpcqaIXaqmcqGGSaalcqaIYaGmcqGGSaalcqGGUaGlcqGGUaGlcqGGUaGlcqGGSaalcqWGUbGBcqGG7aWoaeaacqWGQbGAcqGH9aqpcqaIXaqmcqGGSaalcqaIYaGmcqGGSaalcqGGUaGlcqGGUaGlcqGGUaGlcqGGSaalcqWGWbaCcqGHsislcqaIYaGmaaaaaaaa@A45B@

#### Step 5: Combined symbolic pattern

Two matrices F and S are combined column-wise to constitute the final pattern matrix H.

*H*_*n *× (2*p*-3) _= [*F*_*n *× (*p*-1)_|*S*_*n *× (*p*-2)_]

For each gene, a sequence of 2*p *- 3 letters represents its cluster membership.

#### Step 6: Reassigning minor clusters

For better interpretability, we reassigned genes of *minor clusters *– clusters with fewer members than a predefined threshold – to the nearest cluster with the most correlated genes.

#### Step 7: Output

As output, we get a membership list for each gene, and a 2-dimensional symbolic 'pattern map' with the first-order difference pattern on the horizontal axis and the second-order on the vertical axis. In each cell of the pattern map, every member profile is drawn along the time-point axis with error bars of one standard deviation.

### Identifying the number of clusters

We performed quasi-exhaustive searching to determine the optimal number of clusters. We ran DIB-C 1, 296 times varying threshold values from *T *= {1 × 10^-5^, 2 × 10^-5^, ..., 9 × 10^-5^, ..., 1 × 10^-2^, ..., 9 × 10^-2^}, where |*T*| = 1, 296. The clustering number which maximized the Z-score for the real data was chosen as the optimal clustering number. Since GO IDs were not available for the simulated data, an APF-maximizing threshold was used instead.

### Visualization

DIB-C provides a Directed Acyclic Graph (DAG) representation of multiple clustering results obtained at different threshold levels. Multiple clustering results are used to construct a DAG. Each node represents a symbolic pattern of *difference *and each edge depicts the parent-child relationship between two nodes. The root-node is always an all-null pattern, irrespective of the threshold. Next, we obtain the first clustering result in level 1 from the smallest threshold, the third clustering result in level 2 from the second smallest threshold, and so on until all thresholds were used up. Clusters in the previous layer defined by a smaller threshold are repartitioned in the next layer defined by a larger threshold. Hence, the number of clusters increases or stays the same as the threshold number increases. In each level, nodes with a common first-order pattern have the same color. Clicking on the node leads to a detailed profile of all member genes in a single cluster with error bars.

Once a final level (or the corresponding final result of clustering) is chosen, the clusters in that level are reorganized into a 2-dimensional pattern map. The columns represent the first-order difference, and the rows represent the second-order difference. Each cell represents one symbolic pattern and contains a detailed profile of all members, with error bars.

## Authors' contributions

JK conceived of the algorithm and carried out the data analysis. JHK oversaw the research and contributed to the evaluation procedure and the visualization. All authors contributed to preparation of the manuscript.
